# Design and experiment of spiral conveying pipe in pneumatic centralized fertilizer discharge system

**DOI:** 10.1371/journal.pone.0320126

**Published:** 2025-06-24

**Authors:** Longmei Zhang, Wensheng Yuan, Yugang Feng, Chengqian Jin, Gangwei Liu, Shuangcheng Xie

**Affiliations:** Nanjing Institute of Agricultural Mechanization, Ministry of Agriculture and Rural Affairs, Nanjing, China; China University of Mining and Technology, CHINA

## Abstract

To address the problem of poor fertilizer uniformity caused by vibration and skewing during rice transplanter operations, this study developed a pneumatic centralized spiral fertilizer discharge system. By leveraging the combined effects of high-speed swirling airflow and a spiral conveying pipe, the system generates a high-speed rotating air–fertilizer mixed flow, mitigating the negative effects of ground unevenness and machine vibration on fertilizer performance. Through multi-parameter coupled simulation experiments, the optimal working parameters for the spiral conveying pipe were identified as follows: a spiral pipe length of 444.35 mm, a cross-sectional slope angle of 26°, an airflow velocity of 35 m s^−1^, and a screw pitch of 105 mm, achieving a coefficient of variation of 4.61%. To simulate complex field environments, comparative bench experiments were conducted between the spiral conveying pipe and the smooth straight pipe. The results showed that, at inclination angles of 0°, 5°, 10°, and 15°, the coefficients of variation for the spiral conveying pipe were 4.53%, 5.87%, 8.47%, and 9.64%, respectively, significantly outperforming the smooth straight pipe. Compared to the smooth straight pipe, the spiral conveying pipe reduced the coefficients of variation by 50.81%, 54.07%, 44.53%, and 50.54%, respectively. Field experiments demonstrated that the coefficient of variation for the spiral conveying pipe was 5.27%, representing a 63.1% reduction compared to the 14.28% recorded for the smooth straight pipe. The results confirm the effectiveness of the spiral conveying pipe’s structural design and its superior fertilizer performance, making it highly suitable for complex paddy field environments.

## Introduction

Fertilizers provide essential nutrients for crop growth and are a key factor in improving crop yields [1,2]. Enhancing the efficiency and precision of fertilizer application, as well as ensuring uniform distribution is essential for ensuring high-quality and high-yield grain production [3–5]. The pneumatic centralized fertilizer discharge system uses high-speed airflow to deliver fertilizers to a mixing device, where they are thoroughly mixed with air to form an air–fertilizer two-phase flow. This mixture is then evenly distributed to each row of crops through a distribution mechanism, facilitating precise, multi-row fertilization. This approach offers the advantages of pneumatic transportation and accurate fertilizer application, ensuring an efficient and uniform nutrient supply to crops [6–11].

Currently, research on pneumatic centralized fertilizer discharge systems mainly focuses on components such as fertilizer metering devices [12–15], air–fertilizer mixing devices [16,17], and distribution devices [18–21]. However, limited attention has been paid to the internal structure of the delivery tubes at the lower end of the distributor. The delivery tube is a critical component linking the pneumatic distribution device to the gas-fertilizer mixing device. It is essential for inducing turbulence in the air–fertilizer two-phase flow prior to its entry into the distributor, thereby preventing the accumulation and blockage of fertilizer particles within the tube. This ensures that fertilizer particles flow smoothly and evenly into the distributor and promotes the uniform distribution of the air–fertilizer two-phase flow [22,23]. The commonly used internal structures of delivery tubes include smooth straight tubes and corrugated tubes. Smooth straight tubes are simple in design but often result in fertilizer particles accumulating on the tube walls, causing blockages. While corrugated tubes have been extensively studied, their mixing efficiency and fertilizer delivery capacity remain suboptimal [24–26]. Therefore, further research on the structural forms of delivery tubes is necessary. By optimizing the internal structures of the tubes, the uniformity and stability of fertilizer application rates across rows in the pneumatic centralized fertilizer discharge system can be significantly improved.

To overcome challenges such as uneven fertilizer distribution and inconsistent application rates across rows caused by undulating ground and machinery vibrations in pneumatic centralized fertilizer discharge systems, this study proposes a spiral delivery tube. By combining the characteristics of linear and rotational motion, the air–fertilizer mixture is guided upward along a spiral trajectory. This promotes a more uniform mixing of the air–fertilizer two-phase flow, thereby improving the reliability and stability of the fertilizer discharge system and ensuring an even supply of fertilizer to crops.

## Overall structure and working principle

### Overall structure

**Fig 1:**
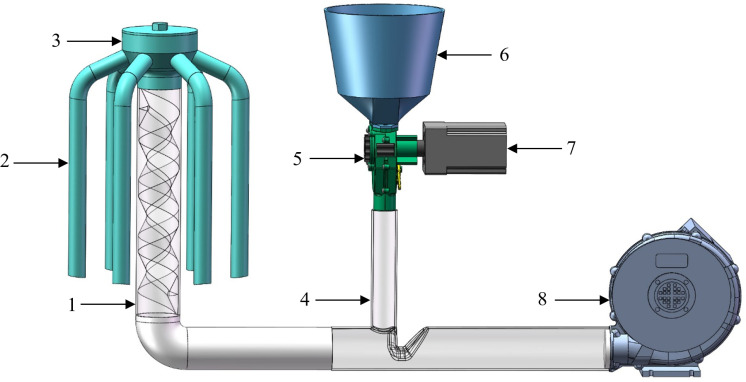
The overall structure of the pneumatic centralized spiral fertilizer discharge system. 1. Spiral conveying pipe 2. Fertilizing pipe 3. Pneumatic distributor 4. Air–fertilizer mixing device 5. Fertilizer discharge device 6. Fertilizer Box 7. Stepper motor 8. Speed-regulating fan.

The pneumatic centralized spiral fertilizer discharge system primarily consists of a fertilizer discharge device, a speed-regulating fan, a spiral conveying pipe, a pneumatic distributor, and multiple fertilizing pipe. The overall structure is shown in Fig 1.

The fertilizer discharge device is powered by a stepper motor and connected to the upper fertilizer inlet of the air–fertilizer mixing device. The air inlet of the air–fertilizer mixing device is connected to the fan, while its outlet is connected to the lower end of the spiral conveying pipe via the air–fertilizer delivery pipe. The inlet of the pneumatic distributor is linked to the upper outlet of the spiral conveying pipe, and each outlet of the pneumatic distributor is connected to a fertilizing pipe.

### Working principle

The pneumatic centralized spiral fertilizer discharge system primarily relies on high-speed airflow and the spiral conveying pipe to create a high-speed rotating air–fertilizer mixture, counteracting the adverse effects of uneven ground and machine vibrations on fertilizer discharge. During operation, fertilizer is first discharged from the tank into the air–fertilizer mixing device by the fertilizer discharge device in a controlled manner. In the air–fertilizer mixing device, the Venturi effect ensures even mixing of high-speed airflow and fertilizer in the mixing chamber. The air–fertilizer mixture then enters the spiral conveying pipe, where the combined effects of the pipe’s inflow, friction, and the reflection of fertilizer particles form the high-speed rotating air–fertilizer mixture. Finally, the pneumatic distributor precisely and uniformly distributes the fertilizer to each outlet, from where it is transported to the soil of each crop row via the fertilizer discharge pipe.

## Design and analysis of spiral conveying pipe

### Calculation of the air flow rate and diameter of the fertilizer delivery pipe

The fertilizer particles achieve a mechanical balance among gravity $F_{g}$, buoyancy $\left(F_{b}\right)$, and resistance (R) in the spiral conveying pipe, maintaining a constant speed while remaining suspended and moving forward through the pipe. The equation of motion for fertilizer particles freely settling under the influence of gravity is given as follows:


$$\begin{array}{c} \left\{ \begin{array}{c} F=F_{g}-F_{b}-R=m\frac{d_{u}}{d_{t}}\\ F_{g}=mg=V\rho_{p}g\\ F_{b}=m\frac{\rho }{\rho_{p}}\cdot g=V\rho g\\ R=CA\left(\frac{\rho u^{2}}{2}\right) \end{array}\right.\; \end{array}$$ (1)

$$\;m\frac{d_{u}}{d_{t}}=V\rho_{p}g-V\rho g-CA\frac{\rho u^{2}}{2}=V\left(\rho_{p}-\rho \right)-CA\frac{\rho u^{2}}{2}$$ (2)

In the formula, R represents the resistance experienced by the particle in the fluid., N; $F_{g}$ represents the gravity of the particle, N; $F_{b}$ represents the buoyancy force experienced by the particle, N; u represents the relative velocity between particles and fluid, m s^−1^; A represents the projected area of the particle in the direction of the fluid motion, m^2^; m represents the mass of the particles, kg; V represents the volume of the particles, m^3^. When the fertilizer particles reach a state of free suspension, substitute $\frac{d_{u}}{d_{t}}=0$, into equation (2),


$$u=v_{p}=\sqrt{\frac{2V\left(\rho_{p}-\rho \right)}{3C\rho }}$$ (3)

Fertilizer particles are irregular spheres, requiring the use of a correction system for irregularly shaped materials. The free suspension velocity of fertilizer particles is determined as follows:


$$v_{p}=\frac{1}{\sqrt{K_{s}}}\cdot \sqrt{\frac{4g\left(\rho_{p}-\rho \right)d_{p}}{3C\rho }}$$ (4)

In the formula, C represents the drag coefficient; $K_{s}$ represents the correction factor for irregularly shaped materials, with a value of 1.2; $\upsilon_{p}$ represents the free suspension velocity of particles, m s^−1^; g represents the acceleration due to gravity, with a value of 9.81m s^−2^; ρ represents the density of the fluid, kg m^−3^; $\rho_{p}$ represents particle density, kg m^−3^; $d_{p}$ represents the equivalent diameter of the particle, m.

The drag coefficient is determined by the Reynolds number of the fertilizer particles and is a function of their Reynolds number relative to the fluid. relative to the fluid. The functional relationship is expressed as follows:


$$C=f\left({\text{Re}}_{p}\right)$$ (5)

$$\begin{array}{c} \left\{ \begin{array}{cc} C=\frac{24}{{\text{Re}}_{p}}\;\;\;\;\;\;\;\;\;\;\;\;\;\;\;\;\;\;\;\;\;\;\;\;\;\; & \;{\text{Re}}_{p}<1\\ C=\frac{24}{{\text{Re}}_{p}}\left(1+{0.15\text{Re}}_{p}^{0.687}\right)\;\; & 1<{\text{Re}}_{p}<1000\\ C=0.44\;\;\;\;\;\;\;\;\;\;\;\;\;\;\;\;\;\;\;\;\;\;\;\;\;\; & \;{\text{Re}}_{p}>1000 \end{array}\right.\; \end{array}$$ (6)

$${\text{Re}}_{p}=\frac{\rho_{g}\left|v_{g}-v_{p}\right|d_{p}}{\mu_{g}}$$ (7)

In the formula, ${\text{Re}}_{p}$ represents the particle Reynolds number; $\mu_{g}$ represents the kinematic viscosity of air, Pa. s; $\upsilon_{g}$ represents the airflow speed, m s^−1^.

Under normal temperature and pressure conditions, the air density is approximately 1.225 kg m^−3^, and the average equivalent diameter of fertilizer particles measured in the experiment is 3.40 mm. At a pressure of 101.325 kPa and a temperature of 20°C, the dynamic viscosity of air is 1.7894×10^−5^ Pa·s, and the difference between the airflow velocity and particle velocity ranges from 5–30 m s^−1^. Substituting these parameters into formula (7) yields particle Reynolds numbers exceeding 1000, indicating a drag coefficient of C=0.44.

The bulk density of the fertilizer particles is 1540 kg m^−3^. By substituting the above parameters into formula (4), the free suspension velocity of the fertilizer particles is calculated to be approximately 10.29 m s^−1^. Considering the complex pipeline structure of the pneumatic centralized spiral fertilizer discharge system, the airflow velocity is set at 2–4 times the suspension velocity of the fertilizer particles, resulting in an airflow velocity range of 25–40 m s^−1^.

The fertilization rate of the fertilizer discharge device is expressed as follows:


$$V_{a}=\frac{u_{a}\cdot l_{a}\cdot m_{a}}{3600}$$ (8)

In the formula, $V_{a}$ represents the fertilization rate, kg s^−1^; $u_{a}$ represents the walking speed of the machinery, km h^−1^; $l_{a}$ represents the fertilization width, m; $m_{a}$ represents the amount of fertilizer applied, kg hm^−2^.

Based on the requirements of synchronous side fertilization during rice transplanting, a six rows rice transplanter was selected as the research object. The fertilizer application rate was calculated to range 250–600 kg hm^−2^, the maximum forward speed of the machine was set at 5.94 km h^−1^, and the fertilization width for six rows was 2 m. The maximum fertilization rate per row was calculated to be 0.33 kg s^−1^, with an additional 10% fertilizer allowance, resulting in approximately 0.363 kg s^−1^.

The material to air conveying ratio of fertilizer particles in the pipeline is expressed as follows:


$$\;\lambda =\frac{M_{a}}{M_{s}}$$ (9)

$$M_{s}=\rho_{s}\frac{\pi D^{2}}{4}\upsilon_{s}$$ (10)

In the formula, λ represents the material to air conveying ratio, with a value of 2.9; $M_{a}$ represents the delivery mass of fertilizer particles per unit time, kg s^−1^; $M_{s}$ represents the air quality delivered per unit time, kg s^−1^; $\rho_{s}$ represents air density, 1.225 kg m^−3^; D represents the diameter of the pipeline, m; $\upsilon_{s}$ represents the airflow velocity, m s^−1^.

Based on formulas (9) and (10), the diameter of the conveying pipeline is determined as follows:


$$\;D=\sqrt{\frac{4M_{a}}{\lambda \pi \rho_{s}\upsilon_{s}}}$$ (11)

Based on the calculations, the pipe diameter is approximately 72 mm.

### Design of the mechanical structure of the spiral conveying pipe

In this study, a spiral conveying pipe featuring a unique three-dimensional spiral track was designed. This pipe integrates the characteristics of linear and rotational motion. The spiral shape enables the air–fertilizer mixture to shift position and direction during ascent, generating rotational motion along the track.

**Fig 2:**
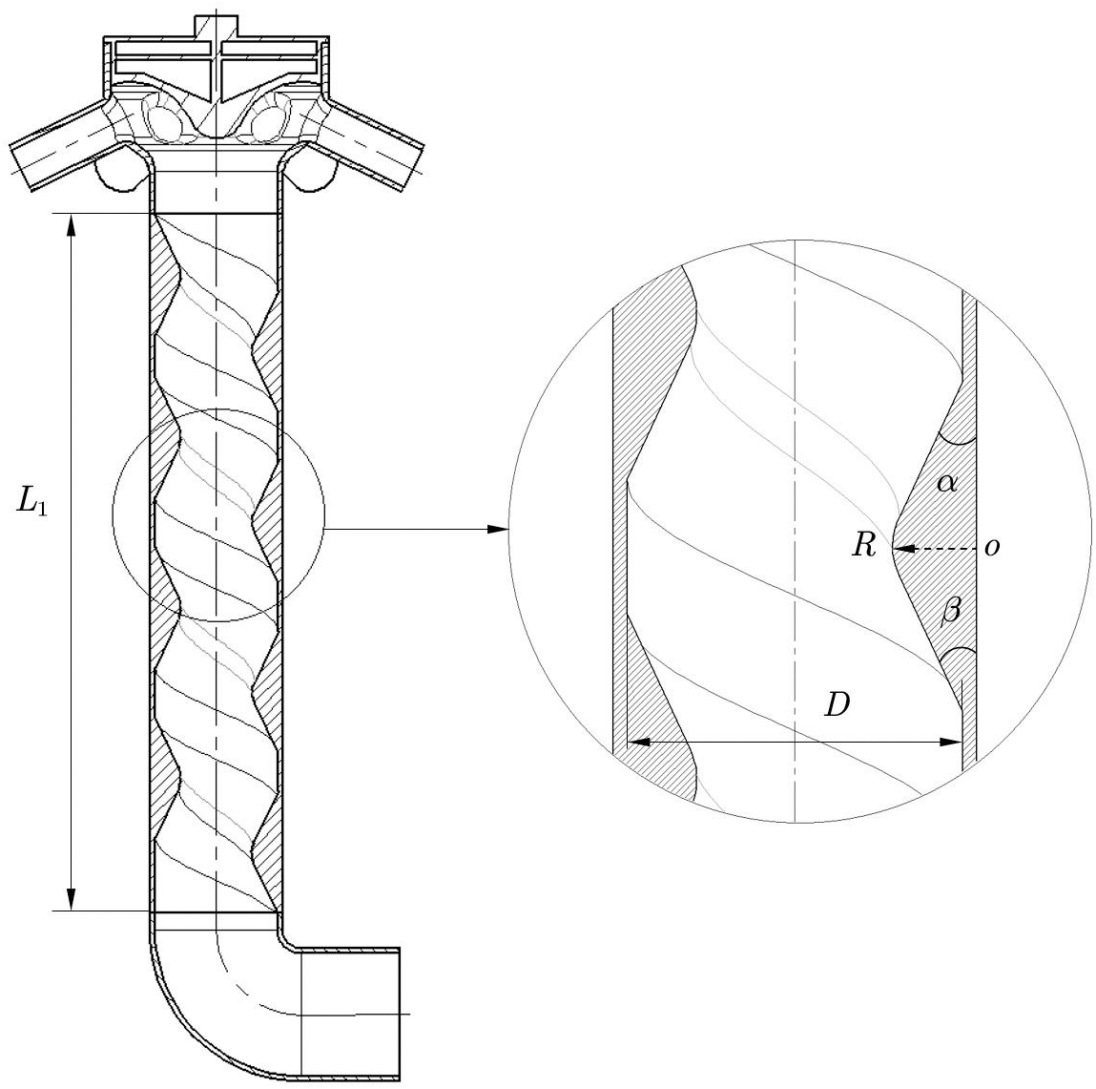
Schematic representation of the mechanical structure of the spiral conveying pipe.

The main geometric parameters of the spiral conveying pipe significantly influence the motion characteristics of the air–fertilizer mixture [27]. These parameters include the length ${\;L}_{1}$, diameter D, pitch of screw P, cross-sectional slope angle α, β and cross-sectional radius R of the spiral conveying pipe, as shown in Fig 2. These parameters determine not only the conveying performance of the spiral conveying pipe but also the fluidity and resistance of the air–fertilizer mixture during transport. The length and diameter of the spiral conveying pipe dictate the capacity and conveying distance of the pipe. Properly designing the length and diameter of the spiral tube ensures that the air–fertilizer mixture maintains a stable flow state during conveyance. The cross-sectional slope angle refers to the angle between the base of the spiral cross-section and the adjacent hypotenuse. The cross-sectional radius is the inferior arc formed by the tangency of the two hypotenuses of the cross-section with a circle of a given radius. A smooth transition reduces the resistance of the air–fertilizer mixture during transport and enhances transportation efficiency. To achieve a smooth transition, a circular arc curve is employed to design the connection between the spiral and the pipeline.

### Analysis of the forces and motion laws governing fertilizer particles in a spiral conveying pipe

Perform a theoretical analysis of the forces and motion patterns of fertilizer particles within the spiral conveying pipe, identifying the factors and conditions influencing their motion characteristics and distribution.

**Fig 3:**
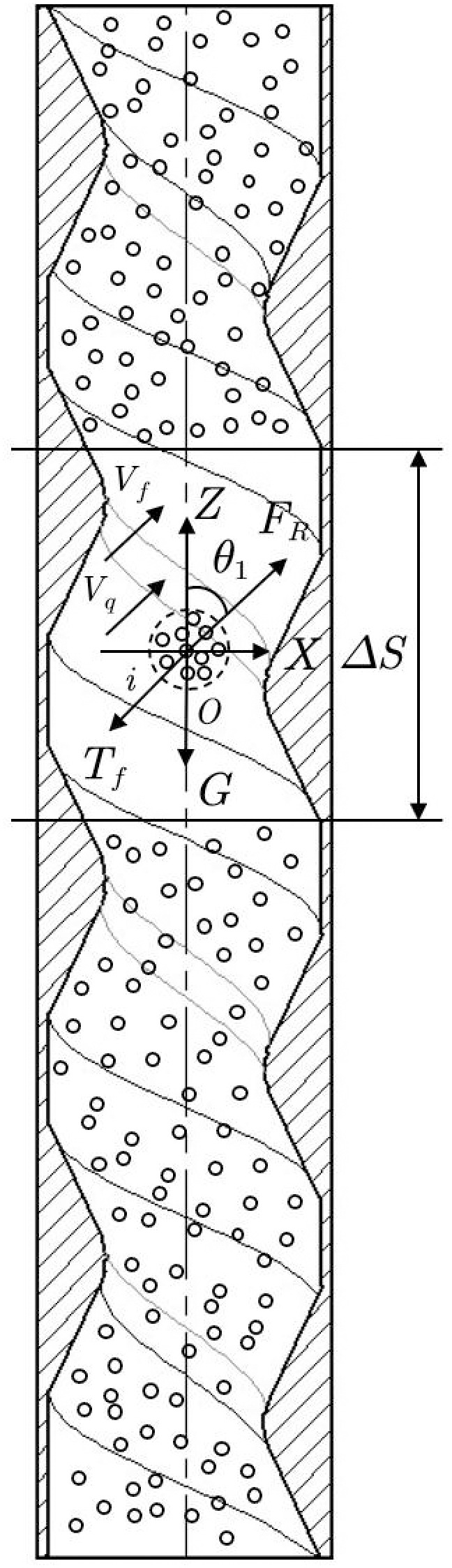
Schematic diagram of force and motion of fertilizer particles. $F_{R}$ represents pneumatic thrust, N; $T_{f}$ represents the pipe wall resistance, N; ΔS represents the selected length of a spiral pipe segment, mm; $\theta_{1}$ represents the helix angle; ${\;V}_{f}$ represents the velocity of fertilizer particles, m s^−1^; $V_{q}$represents the airflow velocity, m s^−1^.


The stress analysis of fertilizer particles within a spiral conveying pipe is illustrated in Fig 3. Using the suspended fertilizer particles in section ΔS of the spiral conveying pipe as the research object, the formula to calculate the aerodynamic thrust acting on the fertilizer particles is as follows:

$$F_{R}=C_{d}A_{1}\rho \frac{{\left(V_{q}-V_{f}\right)}^{2}}{2}$$ (12)

In the formula, $C_{d}$ represents the drag coefficient for flow around; $A_{1}$ represents the projected area of fertilizer particles facing the direction of fluid motion, m^2^; ρ represents the density of air, kg/m^2^.

The formula for calculating the wall resistance $T_{f}$ of the spiral conveying pipe is as follows:


$$T_{f}=\lambda_{1}\rho_{n}A_{2}\frac{\Delta SV_{f}^{2}}{2D}$$ (13)

In the formula, $\lambda_{1}$ represents the drag coefficient; $\rho_{n}$ represents the density of the suspended fertilizer particle group, kg/m^3^; D represents the diameter of the spiral tube, m; $A_{2}$ represents the cross-sectional area of the spiral tube, m^2^.

When the fertilizer particles move in the spiral conveying pipe, according to Newton’s second law, the balance equation among the pneumatic thrust $F_{R}$, pipe wall resistance $T_{f}$, and the gravity ${\;G}_{f}$ of the fertilizer particle group is expressed as follows:


$$\;m\frac{d_{V_{f}}}{d_{t}}=F_{R}-T_{f}-mg\cos\theta_{1}$$ (14)

The differential equation governing the movement of fertilizer particles is as follows:


$$\rho_{n}A_{2}\Delta S\frac{d_{V_{f}}}{d_{t}}=C_{d}A_{1}\rho \frac{{\left(V_{q}-V_{f}\right)}^{2}}{2}-\lambda_{1}\rho_{n}A_{2}\frac{\Delta SV_{f}^{2}}{2D}-mg\cos\theta_{1}$$ (15)

According to formula (15), key factors influencing the movement and distribution of fertilizer particles in the spiral conveying pipe include airflow velocity, spiral pipe diameter, and fertilizer particle velocity.

## Development of simulation models

### Mathematical model for air-solid two-phase flow

The interaction among airflow, fertilizer particles, and the fertilizer discharge system in the pneumatic centralized spiral fertilizer discharge system is highly complex, necessitating the use of the CFD-DEM multi-directional coupling model.

#### Model for fluid motion.

The flow characteristics of the air–fertilizer mixture in the spiral conveying pipe is highly complex. Turbulent vortices are present during the flow process. The Realizablek−epsilon model is used to characterize the fluid motion. This model captures key fluid characteristics in the spiral conveying pipe, such as the generation, development, and dissipation of vortices, as well as the transfer and dissipation of fluid kinetic energy. It provides a detailed description and analysis of the fluid dynamics within the spiral conveying pipe.

#### Model for particle forces.

**Fig 4:**
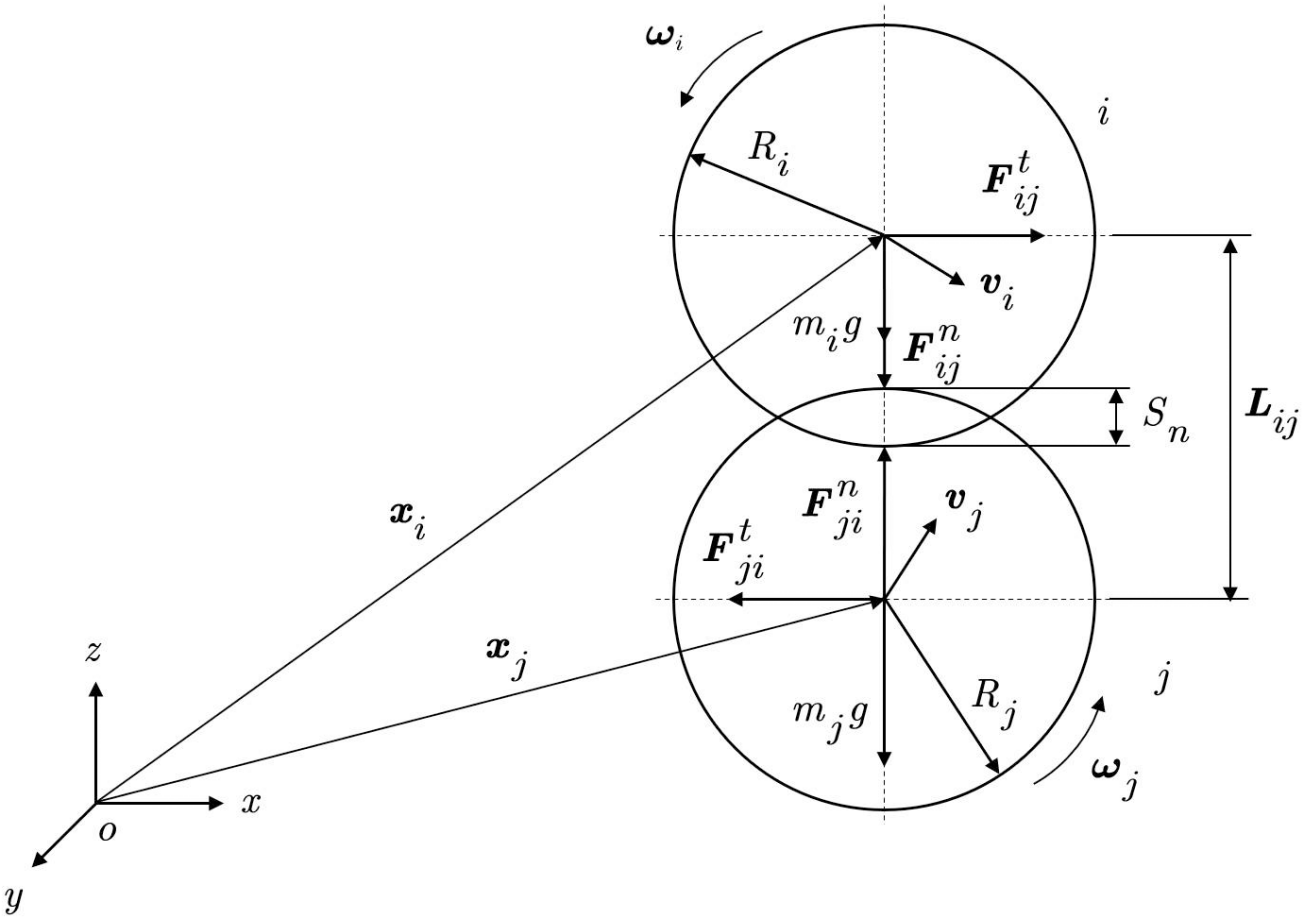
Force analysis during particle collisions.


In the fertilizer discharge system, the interaction forces between fertilizer particles are discontinuous. Therefore, the discrete element method (DEM) is used to analyze the forces acting on the fertilizer particles. The movement of fertilizer particles is governed by contact forces, and the contact algorithm employs a soft contact method that allows a small amount of overlap between particles. Assuming spherical fertilizer particles, the interaction forces, along with their translational and rotational motion, are analyzed, as shown in Fig 4.

$$\begin{array}{c} \left\{ \begin{array}{c} S_{n}=R_{i}+R_{j}-L_{ij}\\ L_{ij}=\left\|x_{j}-x_{i}\right\| \end{array}\right.\; \end{array}$$ (16)

In the formula, Suppose the positions of two particles relative to coordinate system oxyz are $x_{i}$ and $x_{j}$; $R_{i}$, $R_{j}$ represents the radius of particle i, j, m; $L_{ij}$ represents the distance between the centroids of particles, m.


$$\begin{array}{c} \left\{ \begin{array}{c} v_{ij}=v_{i}-v_{j}+n_{ij}\left(R_{i}\omega_{i}+R_{j}\omega_{j}\right)\\ v_{ij}^{n}=n_{ij}\left(v_{ij}n_{ij}\right)\\ v_{ij}^{t}=v_{ij}-v_{ij}^{n} \end{array}\right.\; \end{array}$$ (17)

In the formula, $n_{ij}$ represents a unit vector pointing from the centroid of particle i to particle j; $\omega_{i}$ represents the angular velocity of particle i, rad s^−1^; $v_{ij}$, $v_{ij}^{n}$, $v_{ij}^{t}$ represent the velocity, normal velocity, and tangential velocity during the collision of two particles, m s^−1^.


$$m_{i}\frac{dv_{i}}{dt}=F_{i}^{e}+\sum_{j=1}^{n_{i}^{c}}F_{ij}^{c}+F_{i}^{d}$$ (18)

$$I_{i}\frac{d\omega_{i}}{dt}=T_{i}^{e}+T_{i}^{d}+\sum_{j=1}^{n_{i}^{c}}\delta_{ij}^{c}\times F_{ij}^{c}+\sum_{j=1}^{n_{i}^{c}}T_{ij}^{c}$$ (19)

In the formula, $F_{i}^{e}$ represents the concentrated force applied by the external load to particle i, N; $T_{i}^{e}$ represents the concentrated moment applied to particle i by an external load, *N.M*; $F_{ij}^{c}$ represents the contact force that interacts with neighboring particles and all other elements, N; $T_{ij}^{c}$ represents the contact moment of interaction between adjacent particles and all other elements, *N.M*, $j=1,2,\ldots ,n_{i}^{c}$, $n_{i}^{c}$ represents the number of elements in contact with the particle i; $F_{i}^{d}$ represents the force generated by external damping, N; $T_{i}^{d}$ represents the torque generated by external damping, *N.M*; $\delta_{ij}^{c}$ represents the vector connecting the centroid of particle i and the contact point of element j.

**Fig 5:**
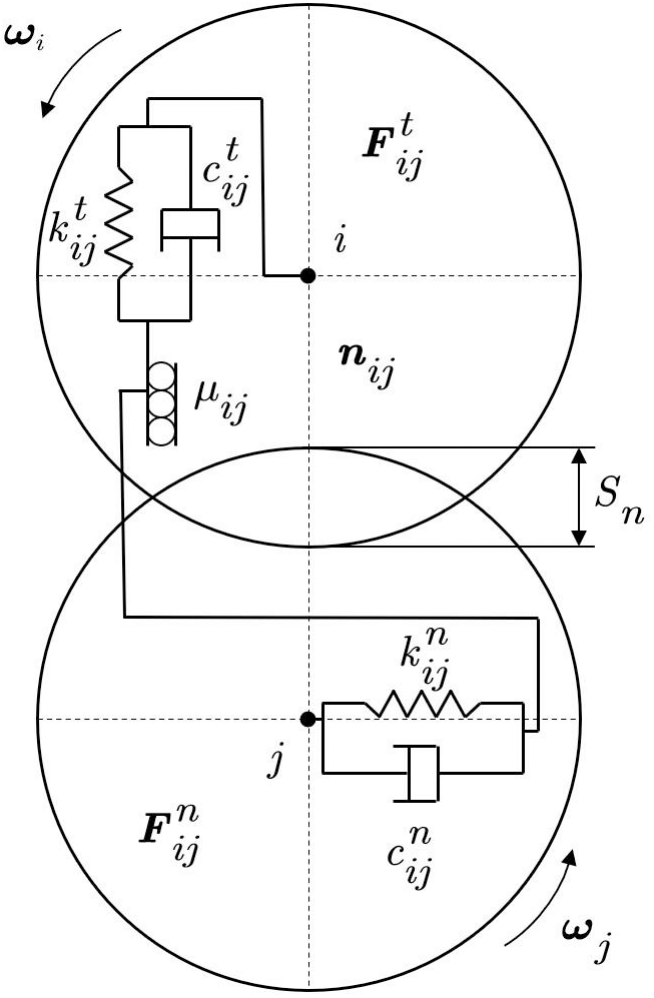
Particle collision model.

When two discrete fertilizer particles collide, slight overlap may occur, resulting in deformation forces. The interaction between two fertilizer particles can be simplified using a spring-damping model, as shown in Fig 5.

The collision force is the resultant force of the normal contact force and the tangential contact force, which can be expressed as follows:


$$F_{ij}=F_{ij}^{n}+F_{ij}^{t}$$ (20)

$$F_{ij}^{n}=\left(-k_{ij}^{n}S_{n}-c_{ij}^{n}S_{n}^{\prime }\right)n_{ij}$$ (21)

$$\begin{array}{c} F_{ij}^{t}=min\left\{ \begin{array}{c} \left(-k_{ij}^{t}S_{t}-c_{ij}^{t}S_{t}^{\prime }\right)t_{ij}\\ -\mu_{ij}\left|F_{ij}^{n}\right|t_{ij} \end{array}\right.\; \end{array}$$ (22)

In the formula, $F_{ij}$, $F_{ij}^{n}$, $F_{ij}^{t}$ represents the resultant force, normal collision force, and tangential collision force, N; $k_{ij}^{n}$ represents the normal elastic coefficient; $k_{ij}^{t}$ represents the tangential elasticity coefficient; $c_{ij}^{n}$ represents the normal damping coefficient; $c_{ij}^{t}$ represents the tangential damping coefficient; $S_{n}$, $S_{t}$ represents the normal and tangential overlap distances, m; $\mu_{ij}$ represents the coefficient of sliding friction between particles; $t_{ij}$ represents a tangential unit vector.

### Simulation test parameters and model of fertilizer particles

#### Simulation model for fertilizer particles.

One hundred randomly selected fertilizer particles were examined to ensure no breakage marks were present, in Fig 7(a). The average length, width, and thickness of the fertilizer particles were measured as 3.61 mm, 3.44 mm, and 3.40 mm, respectively. The average equivalent diameter of fertilizer particles was calculated to be 94.54%.

**Fig 6:**
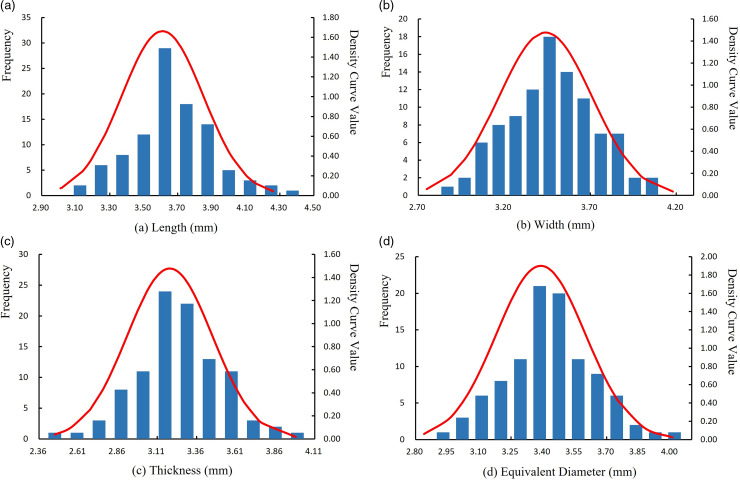
Histogram of fertilizer particle size distribution. (a) length (mm); (b) Width (mm); (c) Thickness (mm); (d) Equivalent diameter (mm).

To visually represent the data distribution of the length, width, thickness, equivalent diameter, and sphericity of fertilizer particles, a histogram with a normal distribution probability density curve was created. The histogram represents the frequency distribution of the geometric dimensions of the fertilizer particle samples, while the probability density curve represents their theoretical distribution. The geometric dimensions of the fertilizer particles were tested for normality. The results of the data analysis are presented in Fig 6.

**Fig 7:**
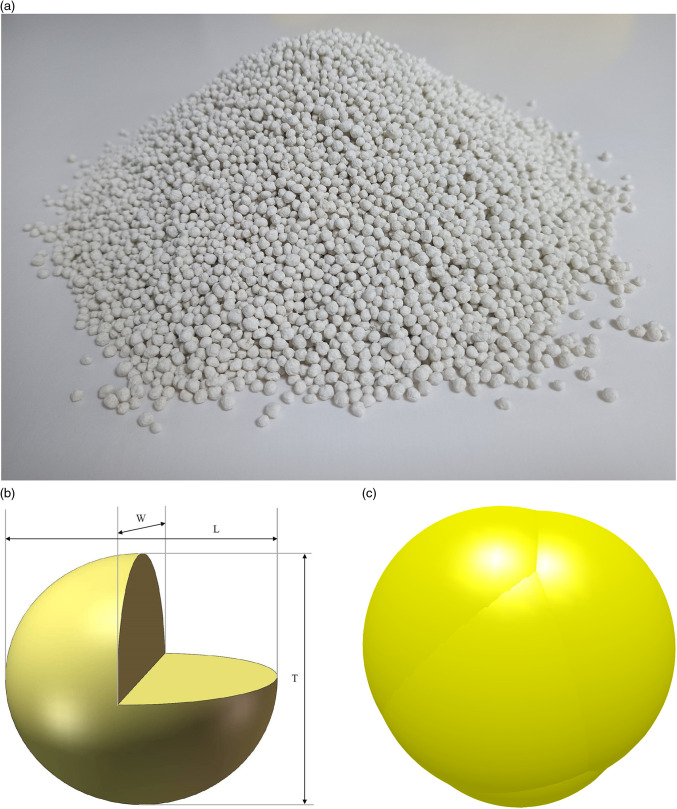
Simulation model of fertilizer particles. (a) Fertilizer particles; (b) Triaxial dimensions; (c) Simulation model.

The three-axis dimensions of the fertilizer particles were measured, in Fig 7(b). A discrete meta-simulation model was developed by filling the irregular fertilizer particles with multi-spherical models, closely approximating the actual particles in both size and shape, as shown in Fig 7(c).

#### Parameters for the coupled simulation experiments.

DEM simulation can model the discrete behavior of fertilizer particles and visually represent their motion in the spiral conveying pipe, including stages such as particle generation, falling, accumulation, conveying, and outflow. The parameters of the DEM simulation experiments are provided in Table 1.

**Table 1:** Parameters for the DEM simulation experiments.

Materials	Parameters	Value
Triaxial dimensions of fertilizer particles	Length × Width × Thickness (mm × mm × mm)	3.61×3.44×3.19
Fertilizer particles	Average diameter (mm)	3.40
	Density (kg m^−3^)	1540
	Poisson’s ratio	0.25
	Young’s modulus (pa)	2.5×10^7^
	Gravitational acceleration (m s^−2^)	9.81
	Elastic modulus (pa)	0.76×10^9^
Tube wall (ABS material)	Density (kg m^−3^)	1060
	Poisson’s ratio	0.394
	Young’s modulus (pa)	2.3×10^9^
	Elastic modulus (pa)	2.6×10^9^
Between fertilizer particles	Coefficient of restitution	0.32
	Coefficient of friction	0.42
	Coefficient of rolling friction	0.13
	Contact model	Hertz-Mind
Between the fertilizer particles and the tube wall	Coefficient of restitution	0.38
	Coefficient of friction	0.29
	Coefficient of rolling friction	0.16
Air	Gravitational acceleration (m s^−2^)	9.81
	Density (kg m^−3^)	1.225
	Viscosity coefficient (Pa·s)	1.789×10^−5^

CFD simulation can divide the fluid domain into fine grids using polyhedron grid division technology, providing deeper insights into fluid motion laws. The parameters of the CFD simulation experiments are presented in Table 2.

**Table 2:** Parameters for the CFD simulation experiments.

Materials	Parameters	Value
Fluid (air)	Gravitational acceleration (m s^−2^)	9.81
	Density (kg m-3)	1.225
	Viscosity coefficient (Pa·s)	1.789×10^−5^
Solid (ABS material)	Density (kg m^−3^)	1060
Velocity inlet	Velocity (m s^−1^)	25–40
Turbulence	Turbulence model	k-epsilon Realizable
	Turbulence intensity (%)	5
	Turbulence viscosity ratio	10
Velocity inlet	Type of inlet	Velocity-inlet
Pressure outlet	Type of outlet	Pressure-outlet
Wall surface	Wall condition	Stationary wall
	shear condition	No slip

### Simulation experiments for parameter optimization

#### Single factor experiments.

**Fig 8:**
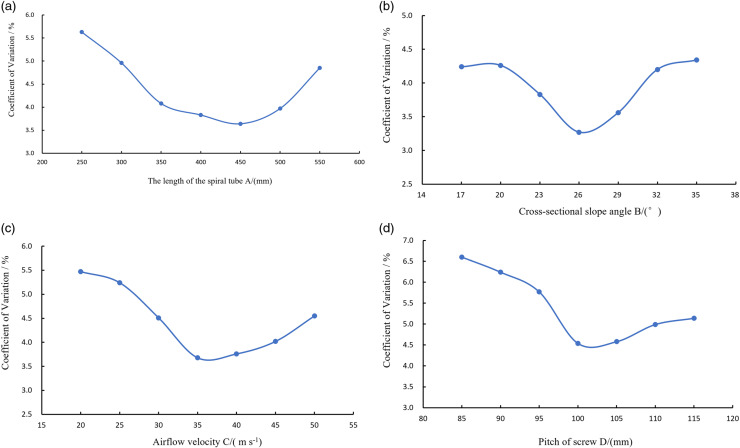
Single factor experiments. A represents the length of the spiral pipe; B represents the cross-sectional slope angle; C represents the airflow velocity; D represents the pitch of screw. (a) B=26°, C=35 m s^−1^, D=105 mm; (b) A=450 mm, C=35 m s^−1^, D=105 mm; (c) A=450 mm, B=26°, D=105 mm; (d) A=450 mm, B=26°, C=35 m s^−1^.

In the single factor test, seven different levels were set for each of the four key factors: the length of the spiral conveying pipe (A), the cross-sectional slope angle (B), the airflow velocity (C), and the pitch of the screw (D), which were tested independently. The test levels for A were: 250 mm, 300 mm, 350 mm, 400 mm, 450 mm, 500 mm, and 550 mm; The test levels for B were: 17°, 20°, 23°, 26°, 29°, 32°, and 35°; The test levels for C were: 20 m s^−1^, 25 m s^−1^, 30 m s^−1^, 35 m s^−1^, 40 m s^−1^, 45 m s^−1^, and 50 m s^−1^; and The test levels for D were: 85 mm, 90 mm, 95 mm, 100 mm, 105 mm, 110 mm, and 115 mm. The test range of A was 250–550mm, the test range of B was 17–35 °, the test range of C was 20–50m s^−1^, and the test range of D was 85–115mm, as shown in Fig 8.

As can be seen from Fig 8, the pneumatic centralized spiral fertilizer discharge system achieves the lowest coefficient of variation in fertilizer application rates across rows when the spiral conveying pipe has a length of 400–500 mm, a cross-sectional slope angle of 23°–29°, an airflow velocity of 35–45 m s^−1^, and a screw pitch of 100–105 mm. This ensures high consistency in fertilizer application.

### Central composite design experiments

#### Response surface simulation experiment design and results.

To explore the impact of the structural parameters of the spiral conveying pipe on the coefficient of variation of discharge consistency across rows, multi-parameter coupling simulation experiments were conducted to analyze the influence of various parameters on discharge uniformity. To enhance discharge consistency across rows in the spiral conveying system, a central composite experimental design was implemented. The factor-level coding table is shown in Table 3, while Table 4 displays the experimental design scheme and results. Each experiment lasted 10 seconds, during which the mass of fertilizer discharged at each outlet was recorded. To compare discharge uniformity across experiments, the coefficient of variation of discharge consistency across rows was calculated, and its average value was determined. The formula for the calculation is as follows:

**Table 3:** Coding of experimental factors.

Code	Experimental factors			
	Length of the spiral conveying pipe A/ (mm)	Cross-sectional slope angle B/ (°)	Airflow velocity C/ (m s^−1^)	Pitch of screw D/ (mm)
-2	300	20	25	90
-1	350	23	30	95
0	400	26	35	100
1	450	29	40	105
2	500	32	45	110

**Table 4:** Experimental scheme and results.

number	A	B	C	D	Coefficient of variation Y/%
1	0	0	0	-2	5.16
2	-1	-1	1	-1	5.52
3	1	-1	-1	-1	3.81
4	-1	1	-1	-1	6.38
5	-1	1	1	-1	4.53
6	-1	-1	-1	1	9.83
7	1	1	-1	-1	7.56
8	0	-2	0	0	7.53
9	1	-1	1	1	5.63
10	1	1	1	-1	6.43
11	-1	-1	-1	-1	6.92
12	-1	1	-1	1	8.47
13	0	0	-2	0	9.87
14	-1	1	1	1	4.12
15	0	2	0	0	5.04
16	0	0	2	0	7.48
17	1	1	1	1	3.14
18	1	-1	1	-1	5.67
19	0	0	0	0	4.65
20	-2	0	0	0	7.54
21	1	1	-1	1	5.68
22	-1	-1	1	1	10.46
23	0	0	0	0	5.34
24	0	0	0	0	5.98
25	0	0	0	0	4.53
26	0	0	0	2	5.27
27	2	0	0	0	5.49
28	1	-1	-1	1	8.75
29	0	0	0	0	5.46
30	0	0	0	0	5.68


$$Y_{1}=\frac{S_{n}}{X_{n}}\times 100\;\%$$ (23)

In the formula, $Y_{1}$ represents coefficient of variation, %; $S_{n}$ represents the standard deviation of displacement for each row, g; $X_{n}$ represents the average displacement of each row, g.

### Development of a regression model and analysis of factor effects

The experimental data were analyzed through multiple regression using Design-Expert 12 software, and the variance analysis results are shown in Table 5.

**Table 5:** Analysis of variance for the regression equation.

Source	Sum of variances	Degree of freedom	Mean square deviation	F	P
Model	86.51	14	6.18	7.99	0.0001^**^
A	7.77	1	7.77	10.05	0.0063^**^
B	9.70	1	9.70	12.55	0.0030^**^
C	11.59	1	11.59	14.99	0.0015^**^
D	3.74	1	3.74	4.84	0.049^*^
AB	4.18	1	4.18	5.41	0.0345^*^
AC	0.2601	1	0.2601	0.3363	0.5706
AD	6.00	1	6.00	7.76	0.0138^*^
BC	3.84	1	3.84	4.97	0.0415^*^
BD	16.48	1	16.48	21.31	0.0003^**^
CD	2.94	1	2.94	3.80	0.0701
A^2^	2.00	1	2.00	2.59	0.1284
B^2^	1.24	1	1.24	1.60	0.2246
C^2^	18.01	1	18.01	23.28	0.0002^**^
D^2^	0.0823	1	0.0823	0.1065	0.7487
Residual	11.60	15	0.7733		
Lack of fit	9.96	10	0.99551	3.03	0.1167
Pure error	1.65	5	0.3290		
Sum	98.11	29			

Note:

* indicates significant difference (p<0.05), and

** indicates extremely significant difference (p<0.01).

The regression equation for the coefficient of variation in discharge consistency across rows of the pneumatic centralized spiral fertilizer discharge system is as follows:


$$\begin{array}{c} Y_{1}=5.27-0.5692A-0.6358B-0.6950C+0.3950D+0.5113AB+\\ 0.1275AC-0.6125AD-0.49BC-1.02BD-0.4288CD+\\ 0.2702A^{2}+0.2127B^{2}+0.8102C^{2}-0.0548D^{2} \end{array}$$ (24)

The model has a p-value of less than 0.01, indicating that it is extremely significant. The lack of fit test for the regression equation yields a p-value of 0.1167, which is not significant, suggesting no other major factors affect the coefficient of variation in displacement consistency across rows. The length of the spiral tube, the cross-sectional slope angle, and the airflow velocity have a highly significant impact on the coefficient of variation, while the screw pitch has a significant impact.

After incorporating the degree of freedom of the insignificant interaction term from Table 5 and the regression equation into the residual term, the regression equation for the coefficient of variation in displacement consistency across rows is as follows:


$$\begin{array}{c} Y_{1}=5.27-0.5692A-0.6358B-0.6950C+0.3950D+0.5113AB\\ -0.6125AD-0.49BC-1.02BD+0.8102C^{2} \end{array}$$ (25)

**Fig 9:**
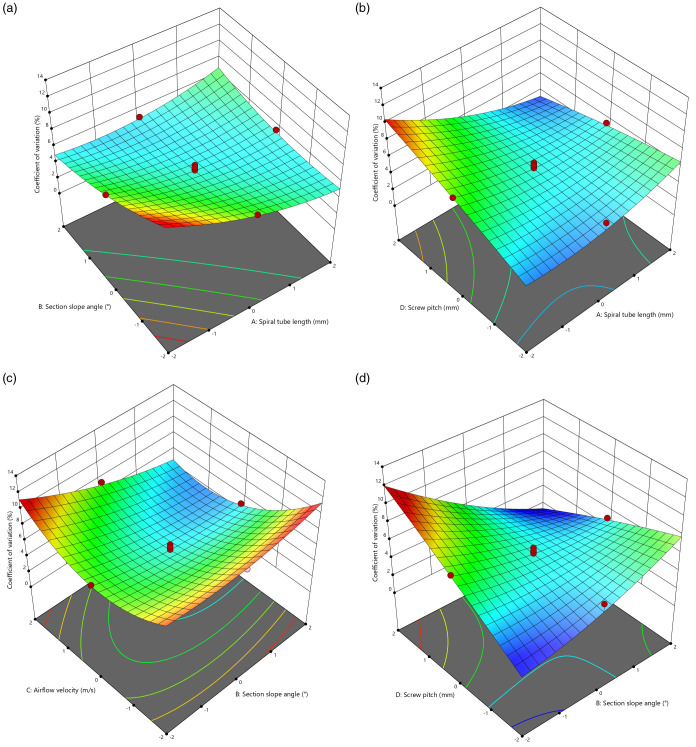
The effect of factor interactions on the coefficient of variation. (a) C=35 m s^−1^, D=105 mm; (b) B=26°, C=35 m s^−1^; (c) A=450 mm, D=105 mm; (d) A=450 mm, C=35 m s^−1^.

The effect of factor interactions on the coefficient of variation is shown in Fig 9. As shown in Fig 9(a), the interaction between A and B on Y shows that when C=35 m s⁻¹ and D=105 mm, with B fixed, Y initially decreases and then increases as A increases. The optimal range for A is 350–450 mm. When A is held constant, Y initially decreases and then increases as B increases, with an optimal range of 20°–29°. As shown in Fig 9(b), the interaction between A and D on Y shows that when B=26° and C=35 m s⁻¹, with A fixed, D is positively correlated with Y. The optimal range for D is 90–105 mm. With D fixed, Y increases as A increases, with an optimal range for A of 350–450 mm. As shown in Fig 9(c), the interaction between B and C on Y shows that when A=450 mm and D=105 mm, with B fixed, Y initially decreases and then increases as C increases. The preferable range for C is 30–40 m s^−1^. With C fixed, Y increases as B increases, with a preferable range for B of 20°–29°. As shown in Fig 9(d), the interaction between B and D on Y shows that when A=450 mm and C=35 m s⁻¹, with B fixed, D is positively correlated with Y. The optimal range for D is 90–105 mm. With D fixed, B is positively correlated with Y, with an optimal range for B of 20–29°.

### Optimization of experimental results

Based on the actual operating conditions and work requirements of the fertilizer discharge system, the constraint conditions for the objective function were selected. The objective function and constraint conditions are as follows:


$$\begin{array}{c} \left\{ \begin{array}{c} minY_{1}(A,B,C,D)\\ S\cdot T\left\{ \begin{array}{c} 350\leq A\leq 450\\ 20\leq B\leq 29\\ 30\leq C\leq 40\\ 90\leq D\leq 105 \end{array}\right.\; \end{array}\right.\; \end{array}$$ (26)

Based on the constraint conditions, the objective function was optimized and solved. Considering the agronomic requirements of actual fertilization operations, the optimal operating parameters of the spiral conveying pipe were obtained: a spiral pipe length of 444.35 mm, a cross-sectional slope angle of 26°, an airflow velocity of 35 m s^−1^, and a screw pitch of 105 mm. To improve the accuracy of the test, five repeated tests were carried out on the spiral conveying pipe under optimal working conditions, and their average value was calculated. The test results showed that the coefficient of variation of displacement consistency was 4.61%, which was very close to the optimization target. Additionally, the error value compared to the optimization target was minimal, verifying the agreement between the spiral conveying pipe’s performance and the optimization result.

**Fig 10:**
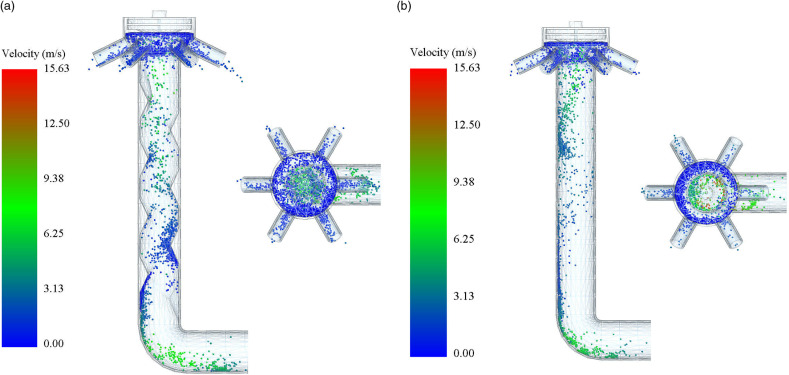
Distribution of fertilizer particles in spiral conveying pipe and smooth straight pipe. (a) Distribution of fertilizer particles in the spiral conveying pipe; (b) Distribution of fertilizer particles in the smooth straight pipe.

To verify the fertilizer discharge performance of the spiral conveying pipe, the motion states of fertilizer particles in the spiral conveying pipe and the smooth straight pipe under the optimal working parameters were compared and analyzed. As shown in Fig 10(a), the fertilizer particles are transported in a spiral conveying pipe in an upward spiral motion, exhibiting smooth flow and uniform mixing. As shown in Fig 10(b), the fertilizer particles move along the inner wall of the smooth straight pipe. The distribution of fertilizer particles is not uniform, and the consistency of fertilizer discharge across rows is poor. Through comparative analysis, it was confirmed that the spiral conveying pipe has better fertilizer discharge performance, indicating the success of the structural optimization.

### Bench experiments

**Fig 11:**
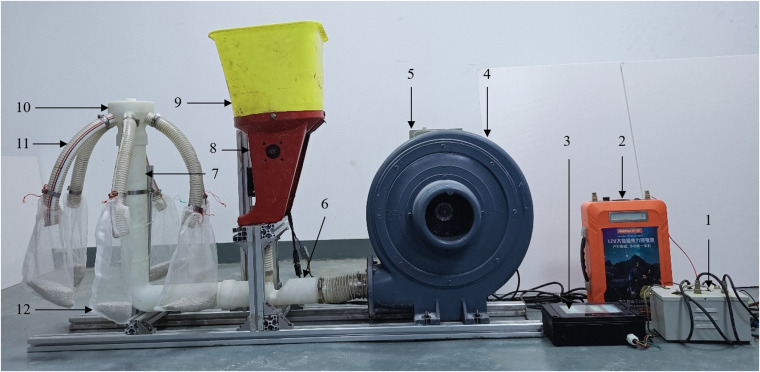
Test bench for the pneumatic centralized spiral fertilizer discharge system. 1. Control box; 2. Battery; 3. Controller; 4. Fan; 5. Frequency converter; 6. Air–fertilizer mixing device; 7. Spiral conveying pipe; 8. Fertilizer discharging device; 9. Fertilizer box; 10. Pneumatic distribution device; 11. Fertilizer conveying pipe; 12. Mesh bag.

To verify the working performance of the spiral conveying pipe under the optimal parameter combination in the simulation experiment, a bench structure was designed based on the optimal simulation parameters. The spiral conveying pipe was 3D printed, and a test bench was set up to conduct bench experiments to assess its feasibility and practicality, as shown in Fig 11.

Compound fertilizer was selected as the test material. First, 10 kg of fertilizer was added to the fertilizer box, and a calibration test of the fertilizer discharge system was carried out. The frequency of the inverter was adjusted to achieve a fan wind speed of 35 m s^−1^, and the motor speed of the fertilizer discharge device was adjusted. When the control system was calibrated, a set of new mesh bags was installed, and the bench test was carried out. The test time was five seconds. The fertilizers collected in the six mesh bags were weighed and recorded. The test was repeated three times. The test data are presented in Table 6.

**Table 6:** Bench comparison experiments.

	Average fertilizer discharge of spiral conveying pipe/ (g)				Average fertilizer discharge of smooth straight pipe/ (g)			
Numbers	0°	5°	10°	15°	0°	5°	10°	15°
Outlet 1	161.38	164.34	169.87	157.69	153.38	168.63	162.35	158.64
Outlet 2	152.76	159.67	157.63	155.53	146.53	150.23	147.26	149.67
Outlet 3	166.83	151.18	143.56	142.86	158.54	136.45	128.37	118.64
Outlet 4	159.67	153.26	152.63	149.78	159.65	143.65	136.47	129.97
Outlet 5	176.64	177.91	181.75	184.68	185.98	189.67	191.63	204.63
Outlet 6	167.53	171.98	178.86	181.26	184.87	187.65	188.74	192.54
Average value	164.14	163.06	164.05	161.97	164.83	162.71	159.14	159.02
Standard deviation	7.44	9.58	13.89	15.61	15.17	20.79	24.30	31.00
Coefficient of variation/ (%)	4.53	5.87	8.47	9.64	9.21	12.78	15.27	19.49

**Fig 12:**
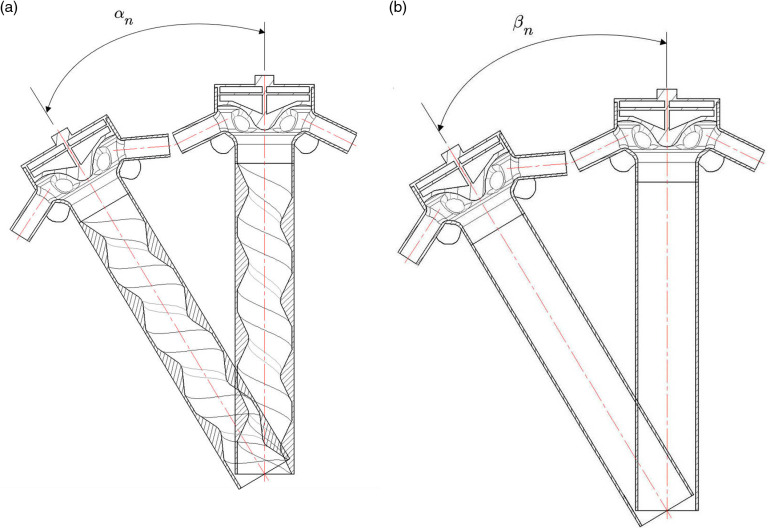
Comparative experiments. (a) Inclination angle of the spiral conveying pipe; (b) Inclination angle of the smooth straight pipe.

To simulate the effects of ground undulations, as well as vibrations and deflections in agricultural machinery, on the consistency of discharge volume across rows, a comparative experiment was conducted using a spiral conveying pipe and a smooth straight pipe, as shown in Figs 12(a) and 12(b). Bench tests were performed on the spiral conveying pipe at inclination angles $\left(\alpha_{n}\right)$ of 0°, 5°, 10°, and 15°, and on the smooth straight pipe at inclination angles $\left(\beta_{n}\right)$ of 0°, 5°, 10°, and 15°. The test data are shown in Table 6.

When the inclination angles of the spiral conveying pipe were set to 0°, 5°, 10°, and 15°, the coefficients of variation for fertilizer distribution consistency across rows were 4.53%, 5.87%, 8.47%, and 9.64%, respectively. In comparison, at the same inclination angles, the coefficients of variation for fertilizer distribution consistency for the smooth straight pipe were 9.21%, 12.78%, 15.27%, and 19.49%, respectively. At inclination angles of 0°, 5°, 10°, and 15°, the coefficient of variation for fertilizer discharge consistency was reduced by 50.81%, 54.07%, 44.53%, and 50.54%, respectively, compared to the smooth straight pipe. These results demonstrate that the spiral conveying pipe provides significantly improved fertilizer distribution uniformity compared to the smooth straight pipe across varying inclination angles. This confirms the effectiveness of the spiral conveying pipe’s structural design in improving fertilizer application consistency across rows.

### Field experiments

**Fig 13:**
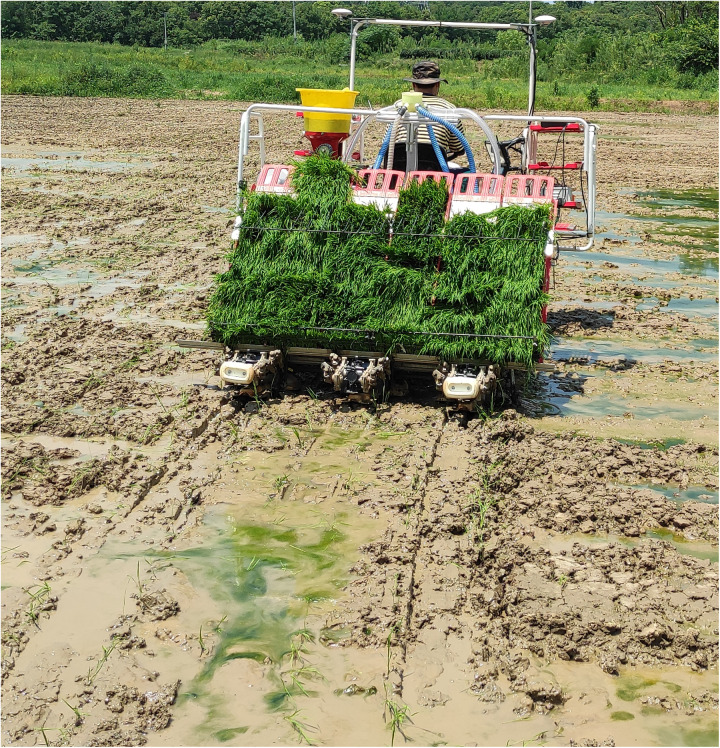
Field experiments.

To test the fertilizer discharge performance of the spiral conveying pipe, field experiments were performed. The experimental prototype selected was a seated high-speed rice transplanter, featuring a fertilization width of 2 m for six rows and a row spacing of 30 cm. Additionally, a pneumatic centralized spiral fertilizer discharge system was installed on the rice transplanter. High standard farmland was selected for field experimentation. The experimental field measures 54 meters in length and 26 meters in width. Prior to the experiment, tillage and land preparation were conducted to ensure that the field surface is level and clean, with elevation differences not exceeding 3 cm, mud depth not surpassing 30 cm, and a water depth 2–3 cm. The absolute moisture content of the paddy field should be kept within 35%–55%. The field experiment is shown in Fig 13.

After adjusting various parameters, field experiments were conducted. First, 10 kg of fertilizer was added to the fertilizer box, then the fan was activated, and the fertilizer wheel speed was adjusted. The forward speed of the rice transplanter was maintained at 5.4 km h^−1^. The test time was five seconds. Fertilizer was collected from six outlets, and this process was repeated three times to calculate the average value. The test data are presented in Table 7.

**Table 7:** Field experiments.

	Average fertilizer discharge of spiral conveying pipe/ (g)				Average fertilizer discharge of smooth straight pipe/ (g)			
Number	1	2	3	Average value	1	2	3	Average value
Outlet 1	163.64	173.38	147.56	161.53	199.75	176.36	165.34	180.48
Outlet 2	153.67	167.89	167.54	163.03	186.26	167.89	157.64	170.60
Outlet 3	149.63	159.36	176.84	161.94	158.67	163.25	131.35	151.09
Outlet 4	154.63	153.38	170.45	159.49	151.36	125.56	138.76	138.56
Outlet 5	174.43	151.68	167.89	164.67	129.45	144.67	203.46	159.19
Outlet 6	168.17	164.54	160.68	164.46	145.18	187.16	184.98	172.44
Average value	160.70	161.71	165.16	162.52	161.78	160.82	163.59	162.06
Standard deviation	8.77	7.72	9.20	8.56	24.08	20.39	24.97	23.15
Coefficient of variation/ (%)	5.46	4.78	5.57	5.27	14.89	12.68	15.27	14.28

Field experiments revealed that the coefficient of variation for the average fertilizer amount per row was 5.27% for the spiral conveying pipe and 14.28% for the smooth straight pipe. Compared to the smooth straight pipe, the spiral conveying pipe increased fertilizer discharge uniformity by 63.1%, indicating superior performance in complex paddy field environments.

## Conclusions

(1) A spiral conveying pipe was designed to address issues such as uneven fertilizer distribution, inconsistent displacement across rows, and excessive, insufficient, or missing fertilization in complex paddy field environments. The pipe generates a high-speed rotating air–fertilizer mixed flow. Theoretical analysis of the forces and movement patterns of fertilizer particles within the spiral conveying pipe led to the establishment of force and fluid movement models. Single-factor tests and simulation experiments identified the optimal range for key structural parameters of the spiral conveying pipe. These parameters include a spiral pipe length of 400–500 mm, a cross-sectional slope angle of 23°–29°, an airflow velocity of 35–45 m s^−1^, and a screw pitch of 100–105 mm.
(2) To explore the impact of structural parameters of the spiral conveying pipe on the coefficient of variation, multi-parameter coupling simulation experiments were conducted. Central composite design experiments were performed to develop a regression equation describing the effect of each factor on the coefficient of variation. Response surface and optimization analysis methods, based on simulation experiment results, identified the optimal working parameters for the spiral conveying pipe: a spiral pipe length of 444.35 mm, a cross-sectional slope angle of 26°, an airflow velocity of 35 m s^−1^, and a screw pitch of 105 mm.
(3) The performance of the spiral conveying pipe in the pneumatic centralized fertilizer system was assessed through simulation, bench, and field experiments. The results revealed that under optimal structural parameters, the coefficients of variation for fertilizer distribution consistency were 4.61%, 4.53%, and 5.27%, respectively, demonstrating high stability and reliability. At inclination angles of 0°, 5°, 10°, and 15°, the spiral conveying pipe achieved significantly greater fertilizer discharge uniformity than the smooth straight pipe, with reductions in the coefficient of variation of 50.81%, 54.07%, 44.53%, and 50.54%, respectively. Field experiments showed that the coefficient of variation for fertilizer discharge consistency for the spiral conveying pipe was 5.27%, representing a 63.1% reduction compared to the 14.28% recorded for the smooth straight pipe. These results confirm the effectiveness of the spiral conveying pipe’s structural design and its superior performance in ensuring fertilizer distribution uniformity in complex paddy field environments.
